# Mesenchymal Stromal Cell-Derived Extracellular Vesicles Pass through the Filtration Barrier and Protect Podocytes in a 3D Glomerular Model under Continuous Perfusion

**DOI:** 10.1007/s13770-021-00374-9

**Published:** 2021-07-27

**Authors:** Linda Bellucci, Giovanni Montini, Federica Collino, Benedetta Bussolati

**Affiliations:** 1 Department of Molecular Biotechnology and Health Sciences, University of Torino, via Nizza 52, 10126 Turin, Italy; 2 Laboratory of Translational Research in Paediatric Nephro-Urology, Fondazione Ca’ Granda IRCCS Ospedale Maggiore Policlinico, Milan, Italy; 3 Department of Clinical Sciences and Community Health, University of Milano, Milan, Italy; 4 Pediatric Nephrology, Dialysis and Transplant Unit, Fondazione Ca’ Granda IRCCS, Policlinico Di Milano, Milan, Italy

**Keywords:** Glomerular permeability, Podocytes, Exosomes, Mesenchymal stromal cell

## Abstract

**Background:**

Dynamic cultures, characterized by continuous fluid reperfusion, elicit physiological responses from cultured cells. Mesenchymal stem cell-derived EVs (MSC-EVs) has been proposed as a novel approach in treating several renal diseases, including acute glomerular damage, by using traditional two-dimensional cell cultures and* in vivo* models. We here aimed to use a fluidic three-dimensional (3D) glomerular model to study the EV dynamics within the glomerular structure under perfusion.

**Methods:**

To this end, we set up a 3D glomerular model culturing human glomerular endothelial cells and podocytes inside a bioreactor on the opposite sides of a porous membrane coated with type IV collagen. The bioreactor was connected to a circuit that allowed fluid passage at the rate of 80 µl/min. To mimic glomerular damage, the system was subjected to doxorubicin administration in the presence of therapeutic MSC-EVs.

**Results:**

The integrity of the glomerular basal membrane in the 3D glomerulus was assessed by a permeability assay, demonstrating that the co-culture could limit the passage of albumin through the filtration barrier. In dynamic conditions, serum EVs engineered with cel-miR-39 passed through the glomerular barrier and transferred the exogenous microRNA to podocyte cell lines. Doxorubicin treatment increased podocyte apoptosis, whereas MSC-EV within the endothelial circuit protected podocytes from damage, decreasing cell death and albumin permeability.

**Conclusion:**

Using an innovative millifluidic model, able to mimic the human glomerular barrier, we were able to trace the EV passage and therapeutic effect in dynamic conditions.

## Introduction

Understanding the uptake and effect of therapeutic agents in dynamic conditions and possibly in three-dimensional models is relevant for their clinical application. At present, preclinical *in vitro* studies usually rely on living cells growing in a dish in the absence of a flow, making the treatment conditions very different from the physiological ones [1]*.* On the contrary, three dimensional (3D) millifluidic systems, composed of living cells seeded in a bioreactor and a continuous fluid perfusion system [2], mimic the organ 3D architecture and offer an alternative solution to the animal experimentation studies [3]. In particular, the* in vivo* cell grown, and differentiation can be better obtained in an organ-on-a-chip. The coupling of the chip with fluidic perfusion allows mimicking the role of vascular organ perfusion.

The glomerulus, the kidney's filtering unit, is a specialized bundle of capillaries contained within the Bowman's capsule. The glomerular filtration barrier is composed of the fenestrated endothelium, the glomerular basement membrane, and the visceral epithelial cells called podocytes. The podocytes help create the filtration slit diaphragm and serve as support to help sustain the integrity of the free-standing capillary loops [4]. The glomerular cells are classical targets of numerous diseases, including immune, metabolic, vascular and malignant disorders [5]*.* However, experimental data are coming mainly from* in vivo* models and do not entirely clarify the mechanisms of glomerular biology and the pathogenesis of these diseases [6].

Numerous advances have been made in applying 3D millifluidic systems to the kidney pathophysiology. Individual components of the nephron, such as proximal tubule, distal tubule/medullary collecting duct, have been successfully mimicked using fluidic devices and making a significant contribution to the understanding of the molecular mechanisms underlying drug toxicity and therapies [7]. Moreover, glomerular fluidic systems have been used to explore the pathological mechanisms of hypertensive nephropathy [8, 9] or to characterize differentiated podocytes obtained from human inducible pluripotent stem cells (iPSCs) [10]. Furthermore, the use of shear stress triggers physiological mechanisms such as the deposition of collagen on the glomerular basement membrane and modulates the pathological mechanisms [9, 10].

Extracellular vesicles from mesenchymal stem/stromal cells (MSC-EVs) have been demonstrated to contain genetic and protein material that can activate several repair mechanisms to ameliorate kidney injury [11]. In literature, there is a substantial number of publications supporting their role in promoting tissue repair and reduce inflammation in different pathological models, including models of acute kidney injury (AKI) [12, 13] and chronic kidney disease (CKD) [14, 15]. However, the effect of EV in dynamic condition under flow has not been studied in detail in the glomerular filtration barrier.

In this study, we developed an innovative technology that recapitulates the human glomerular filtration barrier in a bioreactor to evaluate the effect of MSC-EVs on the GFB under a continuous perfusion flow. The millifluidic dynamic system allowed us to track the fate of different sources of EVs in entering glomerular target cells. Moreover, we evaluate the ability of MSC-EVs infused in the system to protect podocytes from the damage induced by doxorubicin, an anticancer drug with toxic effects on the kidney [16].

## Materials and methods

### Cell lines

A podocyte cell line (h-PODs) obtained as described [17] was used in all the experiments. The cells were cultured in Dulbecco's modified Eagle's medium DMEM High Glucose Euroclone S.p.A., Pero MI, Italy 10% fetal bovine serum (FBS) (Euroclone S.p.A.), with the addition of penicillin–streptomycin (PS) and L-glutamine (Sigma-Aldrich, St. Louis, MO, USA).

Human primary kidney glomerular endothelial cells (h-GECs) were acquired from Cell Biologics (Cell Biologics Inc., Chicago, IL, USA) and subsequently immortalized at passage two by a retrovirus containing p-BABE-puro-hTERT plasmid (Addgene plasmid 1771). Immortalized h-GECs were selected using puromycin (1 µg/ml, Thermo Fisher Scientific, Waltham, MA, USA). The expression of classical endothelial markers was confirmed by flow cytometry analysis using antibodies against CD44, CD144, CD105, CD146, CD31, VE-cadherin, tyrosine-protein kinase receptor Tie-2 (all from Miltenyi Biotec, Bergisch Gladbach, Germany). Cells were expanded in flasks previously coated with attachment factor (Sigma-Aldrich) and cultured in complete EndoGRO-LS Complete Culture Media Kit (Merck Millipore, Burlington, MA, USA) with 10% FBS, PS and MycoZap™ (Lonza, Basel, Switzerland) to prevent contaminations.

MSC-EVs were obtained from Lonza, cultured and characterized as described [18]. MSCs were cultured in Mesenchymal Stem Cells Basal Medium (Lonza) and expanded after 15 days for the first passage and every 7 days for subsequent passages.

### EV isolation

Isolation of EVs from MSCs was performed as described [19]. Confluent MSCs were cultured in serum‐free Roswell Park Memorial Institute medium (RPMI) for about 18 h. The medium was then collected and centrifuged for 30 min at 3000 g to remove cell debris and apoptotic bodies. The supernatant was then ultracentrifuged for 2 h at 100,000 g, 4 °C using the Beckman Coulter Optima L‐100K Ultracentrifuge (Beckman Coulter, Brea, CA, USA) with the rotor type 70 Ti. The EV pellet was resuspended in RPMI supplemented with 1% DMSO and then stored at − 80 °C until further use. EVs were counted using the NanoSight NS300 system (Malvern Instruments Ltd, Malvern, UK). In selected experiments, serum-EVs were used. The serum was centrifuged for 5 min at 3000 rpm at room temperature to eliminate any debris present. The supernatant was subsequently ultracentrifuged at 100,000 for 2 h at 4 °C. EVs pellets were then resuspended in a final volume of 1 ml of RPMI with 1% of DMSO and stored at − 80 °C [20]. EVs were characterized by size, morphology, tetraspanin expression and lack of cytoplasmic protein contamination, as reported in [19, 20].

### Staining of EVs

MSCs were stained with Vybrant™ Cell-labeling Solution (Thermo Fisher Scientific), as described [21]. Briefly, MSCs were detached and resuspended at a density of 1 × 10^6^/mL in a serum-free medium. A cell labelling solution at 5 µl/ mL was added to the cell suspension for 20 min at 37° C. Three washes were then performed with a warm medium by centrifugation at 1500 rpm for 5 min. Cells were then plated, and EVs were collected with the protocol described before. In selected experiments, EVs were directly labelled with 1 μM VybrantTM Cell-labeling Solution during the ultracentrifugation procedure and then washed twice by ultracentrifugation in 1× PBS [22].

### Millifluidic model of the glomerular filtration barrier

A millifluidic device for cell cultures fabricated by IVtech Srl was used (Massarosa LU, Italy). The system allows continuous fluid perfusion (80 µl/min) in a bioreactor where the cells are seeded. The circuit comprises three elements: a peristaltic pump, a bioreactor and two tanks containing liquids passing continuously throughout the system as described [23].

**Fig. 1:**
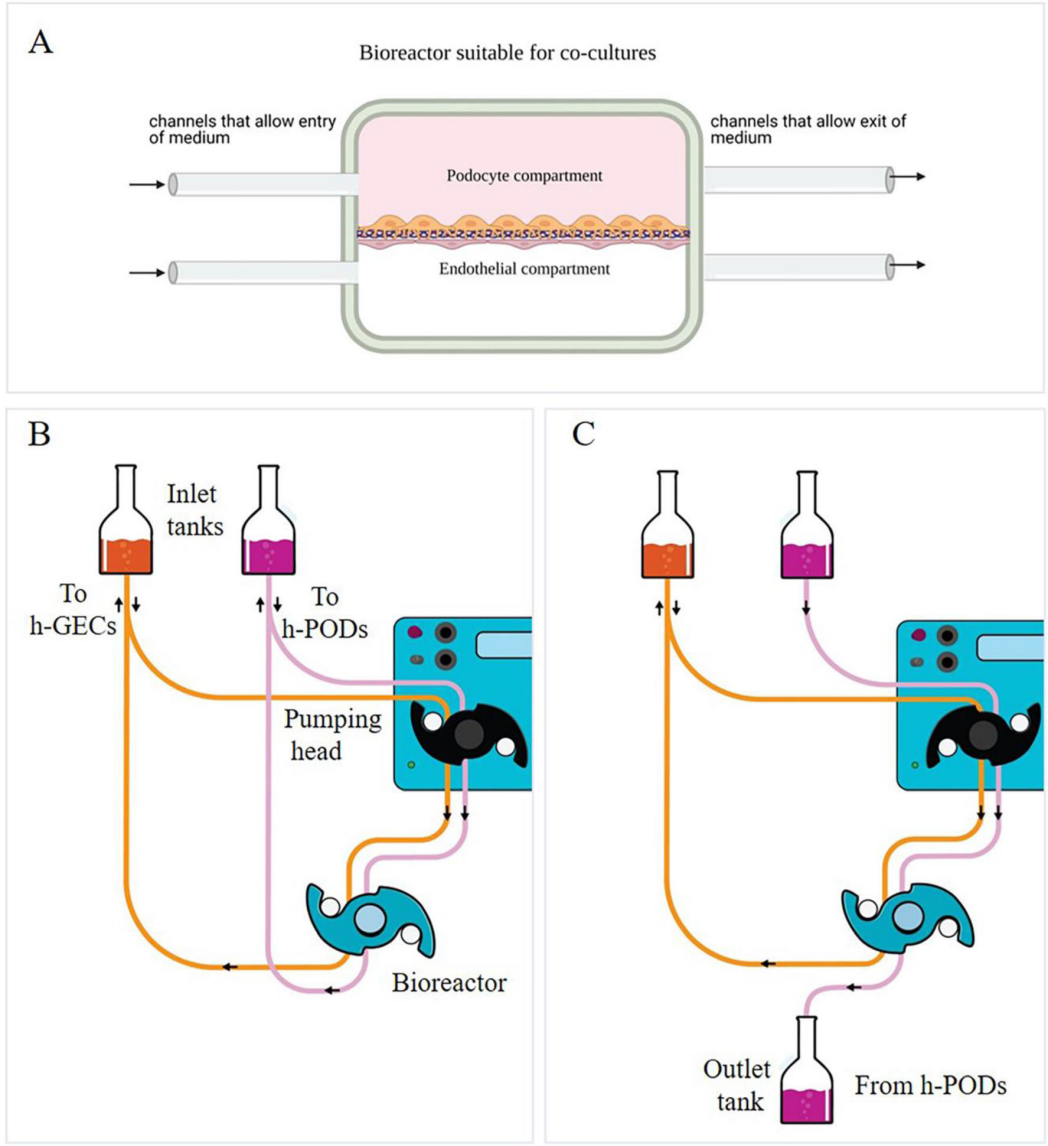
The dynamic glomerular circuit. **A** Schematic representation of the two compartments of the bioreactor in which the h-GEC (bottom) and h-PODs (upper part) were seeded. **B**, **C** Configuration of the dynamic system: constant recirculation (**B**) used in the toxicity study or h-POD fluid collection (**C**) used in the permeability test

Before cell seeding, a porous (Ø 0.45 µl) membrane in PET (ipCELLCULTURE™, it4ip, Louvain-la-Neuve, Belgium) was coated with a solution of type IV collagen (Col IV) (Sigma-Aldrich) diluted in 70% ethanol and incubated overnight in the presence of culture medium. The day after, 1 × 10^5^ h-GEC were seeded on one side of the membrane in 100 µl of the medium. After five hours in the incubator, the bioreactor was assembled, and 1 × 10^5^ h-PODs were seeded on the other side of the membrane (Fig. 1A). After one day, the circuit was connected to allow the fluid flow. The circuit is designed to allow the entry of a cellular stimulus always from the endothelial compartment to mimic factors present in the blood circulation and study their passage through the GFB. Two different flow configurations were used. In the first, the liquid leaving the chambers re-enters in the system in a constant recirculation (Fig. 1B). In the second configuration, used for the permeability test, the culture medium enters one of the chambers and can be collected to the outlet part of the system (Fig. 1C).

### *In vitro* model of acute glomerular damage

A model mimicking acute glomerular damage was achieved by doxorubicin treatment (Sigma-Aldrich). In detail, the endothelial compartment of the dynamic system was filled with DMEM without FBS in the presence of 0.5 µg/ml of doxorubicin, while the h-POD compartment was filled with DMEM w/o FBS. The doxorubicin enriched medium flowed for 3 h in recirculation configuration, at a speed of 80 µl/min. The medium was then changed with basal media supplemented with 2% FBS. In selected experiments, after the doxorubicin treatment, the cells in the bioreactor were washed with PBS, and the basal EndoGRO enriched in growth factors was added in the endothelial compartment in the presence of 4 × 10^9^ MSC-EVs (20.000 EVs per cell) for 24 h with a fluid flow of 80 μl/min.

### Electroporation of EVs with cel-miR-39

To study the uptake of EVs into the podocytes, MSC-EVs were electroporated with an exogenous cel-miR-39 from *C.elegans*. Briefly, 6 × 10^10^ EVs were incubated with 5 µl of cel-miR-39 (20 µM) in 200 µl of the reaction solution (Thermo Fisher Scientific) for 30 min at 37 °C. Electroporation was then carried out with the following program: 10 discharges of 20 ms at 750 V. The EV suspension was then placed at 4 °C overnight. To avoid a non-specific attachment of cel-miR-39 on the cell membranes, MSC-EVs were treated with RNase A (ThermoFisher Scientific), at a concentration of 0.2 mg/mL, for 30 min at 37 °C; then the reaction was stopped using an RNase inhibitor (Thermo Fisher Scientific), according to with manufacturer's protocol. The following day electroporated EVs were ultracentrifuged for 2 h at 100.000 g. The EVs were resuspended in medium and used for uptake studies on the glomerular model. In these experiments, cel-miR-39 alone (20 µM) was introduced into the dynamic system and used as a control.

### Assessment of permeability

Infusion of guanidine isothiocyanate (FITC) conjugated bovine serum albumin (BSA) (Sigma-Aldrich) solution in the endothelial compartment was used to assess barrier integrity. Quantification of the filtration rate through the glomerular filtration barrier was performed [24] for 3 h at the flow rate of 80 ul/min. The configuration used is described in Fig. 1B. The medium enriched in FITC-BSA collected from h-PODs compartment was analyzed in triplicate, with the Promega™ GloMax® Plate Reader (Promega Italia S.r.l., Milano, Italy).

### Evaluation of apoptosis

Apoptotic cell rate was evaluated using the Muse™ Annexin V & Dead Cell Kit (Merck‐Millipore), according to the manufacturer's recommendations. The assay was based on the detection of phosphatidylserine on the surface of apoptotic cells, using fluorescently labelled Annexin V in combination with the dead cell marker, 7- (amino-actinomycin D) AAD. Briefly, after doxorubicin or EV treatments into a dynamic circuit, the cells were detached from the membrane and resuspended in MuseTM Annexin V and a Dead Cell kit. The percentages of the total live cells (negative for Annexin V and dead cell marker) and apoptotic cells (Annexin V^+^ /7-AAD^+^ Annexin V^+^ and 7-AAD^−^) were detected.

### Immunofluorescence studies

The membranes of the dynamic system were removed from the support rings and sectioned. Cells attached to the membrane were fixed in 4% paraformaldehyde (PAF) for 20 min at room temperature (RT) and then permeabilized with 0.1% Triton X100 (Sigma-Aldrich) in PBS 1× for 10 min at 4 °C. Non-specific sites were blocked with 1.5% BSA in PBS 1× for 20 min at RT, followed by incubation with the primary antibodies (Abs) for 1 h at RT. After extensive washes, the secondary Abs and Phalloidin (1: 1000) (Sigma-Aldrich) was added for 1 h at RT. Cell nuclei were stained with 4′,6-diamidino-2-phenylindole (DAPI, 1: 10,000) (Sigma-Aldrich). Finally, coverslips were mounted with Fluoromount-G™ Mounting Medium (Thermo Fisher Scientific). In some cases, to obtain cross-sections of the cells, membranes were included in OCT gel and stored at − 80° C. Transversal cuts at cryotome were then carried out. Imaging was performed using a Leica TCS SP5 confocal system (LEICA Microsystems S.r.l.). Samples were imaged using a 40× PlanApo/1.4 NA oil immersion objectives. Series of x-y-z images (typically 0.19*0.19*0.5 µm^3^ voxel size) were collected along the z-axis at 0.5 µm intervals throughout the sample depth (36 µm). Three-dimensional reconstruction was performed using Image J software.

### PCR analysis

Total RNA was isolated from different cell preparations using Trizol reagent (Ambion, ThermoFisher) according to the manufacturer's protocol. RNA was quantified spectrophotometrically (Nanodrop ND‐1000, Wilmington, DE, USA). The first-strand cDNA was produced from 200 ng of total RNA using the High-Capacity cDNA Reverse Transcription Kit (Applied Biosystems, ThermoFisher). Gene expression analysis was performed by quantitative real-time PCR (qRT-PCR) using glyceraldehyde 3-phosphate dehydrogenase (GAPDH) mRNA as a housekeeping normalizer. The reaction mixes contained 5 ng of cDNA template, sequence-specific oligonucleotide primers (purchased from MWG—Biotech, Eurofins Scientific, Brussels, Belgium) (Table 1) and the Power SYBR Green PCR Master Mix (Applied Biosystems). Fold change expression in respect to control was calculated for all samples.

**Table 1:** List of primers used in qRT-PCR experiments

Gene symbol	Gene	Forward	Reverse
hGAPDH	Glyceraldehyde-3-Phosphate Dehydrogenase	F-CCGCTTCGCTCTCTGCTC	R-CGACCAAATCCGTTGACTCC
hMMP9	Matrix metallopeptidase 9	F-CCGAGCTGACTCGACGGT	R-CCTCGCTGGTACAGGTCGA
hCASP3	Caspase 3	F-CCACAGCACCTGGTTATTATTCTTG	R-AACCCGGGTAAGAATGTGCATA
hCASP7	Caspase 7	F-GAACTTTGATAAAGTGACAGGTATGGG	R-GGCACAAGAGCAGTCATTATAGACAA
hPDX	Podocalyxin	F-CTTGAGACACAGACACAGAG	R-CCGTATGCCGCACTTATC
hSYNPO	Synaptopodin	F-AGCCCAAGGTGACCCCGAAT	R-CCCTGTCACGAGGTGCTGGC

To confirm the presence of cel-miR-39 in the cells, 200 ng of input RNA from all samples were reverse transcribed with the miScript Reverse Transcription Kit and the cDNA was then used to detect cel-miR-39, using the miScript SYBR Green PCR Kit (all from Qiagen, Hilden, Germany). All samples were run in triplicate using 3 ng of cDNA for each reaction described by the manufacturer's protocol (Qiagen). Ct detection cut‐off was set at 35 PCR cycles. In this case, the small nucleolar RNA 6B (RNU6B) was used as a housekeeping normalizer.

All reactions were performed using an Applied Biosystems 7900HT real‐time PCR instrument equipped with a 96-well reaction plate.

### Statistical analysis

Data are shown as mean ± standard deviation (SD). Each experiment was performed at least in triplicate Unpaired t-test or one way ANOVA with Dunnett's multiple comparison test were applied when necessary. All statistical analyses were done with GraphPad Prism software version 8.0 (GraphPad Software, Inc.). *p* values < 0 0.05 were considered significant.

## Results

### Assembly of 3D-glomerular millifluidic model

**Fig. 2:**
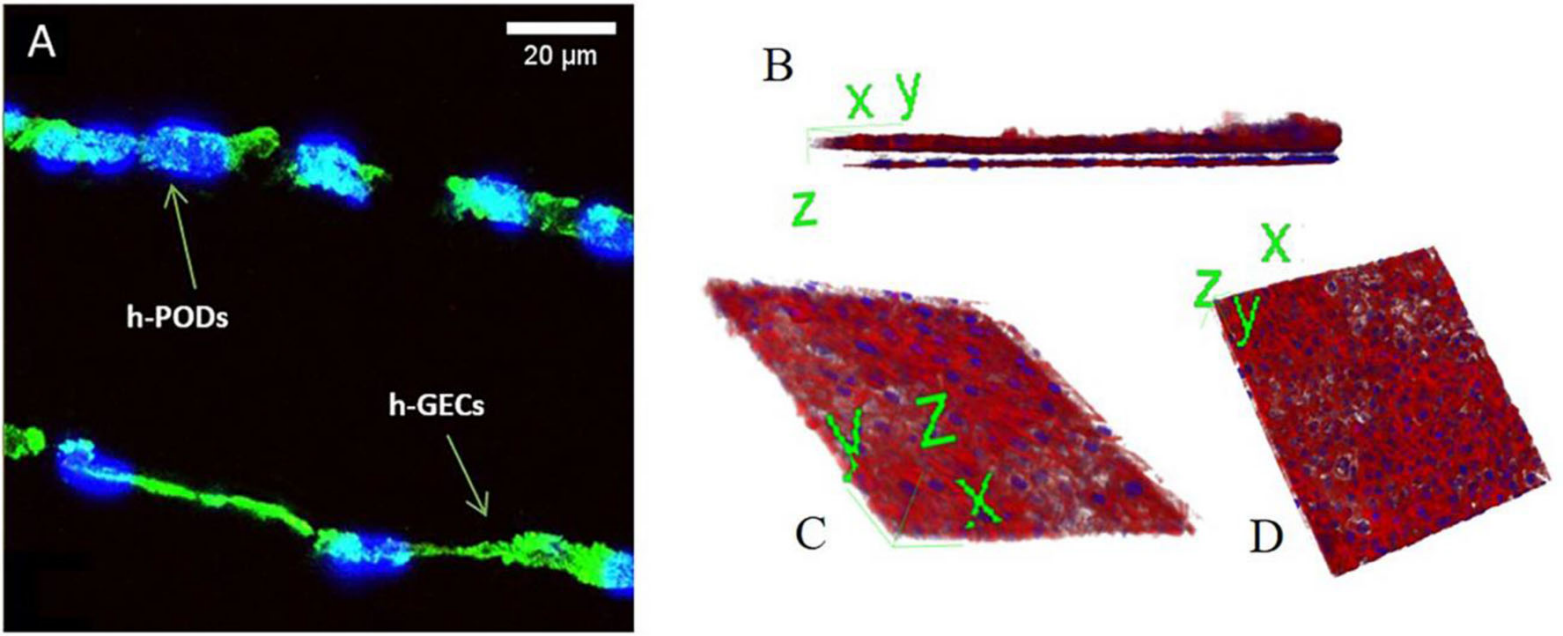
Assembly of dynamic co-cultures. **A** Immunofluorescence staining of the bioreactor membrane in which glomerular cells were grown for 24 h in dynamic condition, fixed and stained for actin (in green) and nuclei (in blue). Cross section of the membranes showed the presence of both h-PODs and h-GECs on the opposite sites of the membrane. **B** 3D reconstruction of the 2 monolayers presents on the opposite side of the membrane. h-PODs were plated on the top and h-GECs on the bottom (actin in red, nuclei in blue). In **C** the podocyte cell monolayer, while in **D** the endothelial monolayer is represented. Images were acquired with the Leica TCS SP5 confocal system confocal microscope, magnification: 40×; scale bar: 20 µm

**Fig. 3:**
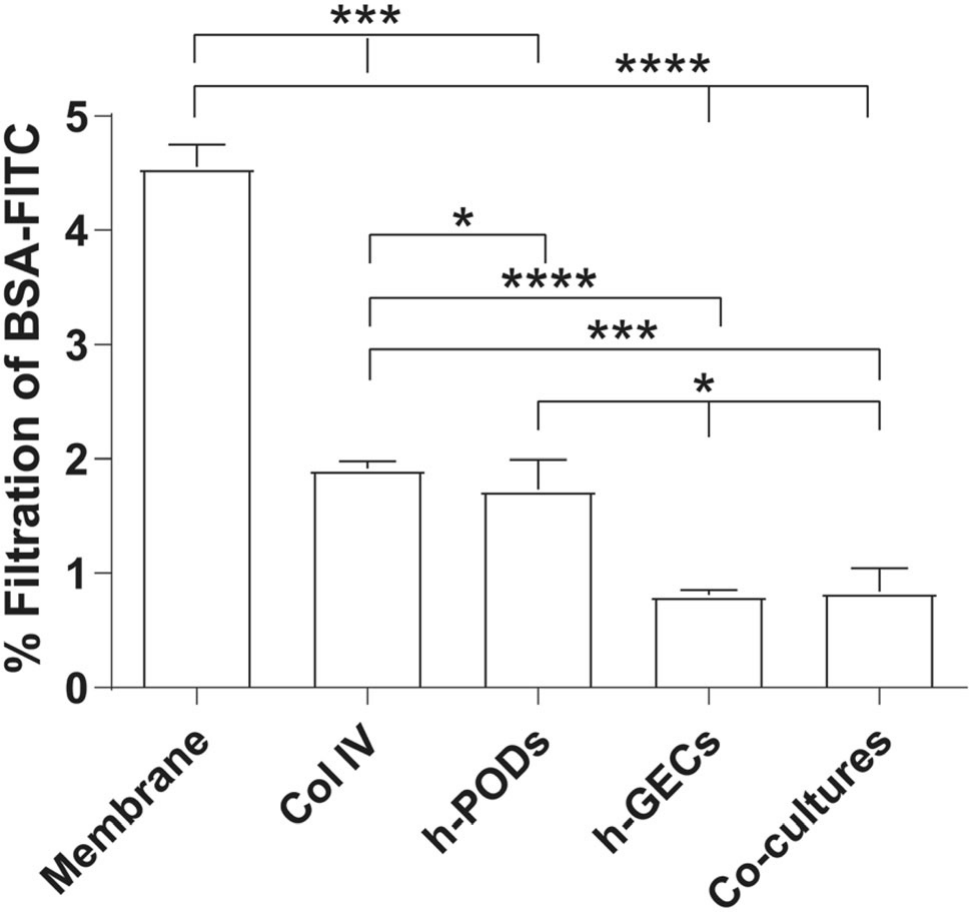
Permeability test for the evaluation of the integrity of 3D-glomerular model. The passage of FITC-BSA through the GFB in the dynamic model was evaluated in 5 different conditions: membranes alone (membrane), membranes coated with type IV collagen (Col IV), and membranes coated with Col IV in presence of h-PODs or h-GECs (h-PODs or h-GECs, respectively) or in presence of the two cells in co-culture (Co-cultures). Perfusion was calculated as a percentage of filtered FITC-BSA (FITC-BSA solution = 1 mg/mL as 100% fluorescence). *p < 0.05, ***p < 0.001, ****p < 0.0001, respectively

A standardized protocol for preparing glomerular co-cultures was obtained, starting from the coating of the porous membrane of a bioreactor with type IV collagen to improve endothelial cell adhesion and ensures the maintenance of the podocyte phenotype [25, 26]. h-GECs and h-PODs were seeded on opposite sides of the membrane, with a density of 100,000 cells/1.9 cm^2^, to allow the formation of uniform cell bilayers. In detail, the endothelial layer was plated in the lower part of the membrane (Fig. 2A), and after 4–5 h required for cell adhesion, the bioreactor was assembled. h-PODs were directly plated through the channel in the upper compartment (Fig. 2A). The next day, the bioreactor was attached to a dynamic circuit equipped with a peristaltic pump, that permits a continuous flux of fluids at the rate of 80 µl/min. Cells were then submitted to different times of perfusion. After 24 h, the glomerular co-culture completely covered the membrane, as shown by the immunofluorescence staining for actin (Fig. 2A) and 3D reconstruction (Fig. 2B–D).

### Assessment of the integrity of the 3D-glomerular model

Since proteinuria is the index of kidney damage, a filtration test based on FITC-BSA was adopted to evaluate the barrier's integrity. Medium enriched with FITC-BSA was flowed into the endothelial circuit for 3 h, mimicking the circulating serum albumin in the bloodstream. We initially tested the capability of different membrane configurations to allow the passage of BSA from the lower (endothelial) to the upper (podocyte) layers. As shown in Fig. 3, the presence of a single component of the glomerular filtration barrier was sufficient to limit the protein filtration in comparison to the uncoated membrane alone. The best results were obtained when the entire co-culture was assembled. Interestingly, the presence of h-GECs was sufficient to strongly reduce the BSA filtration, highlighting the endothelium's contribution to the barrier permeability and to the glomerular structure maintenance.

**Fig. 4:**
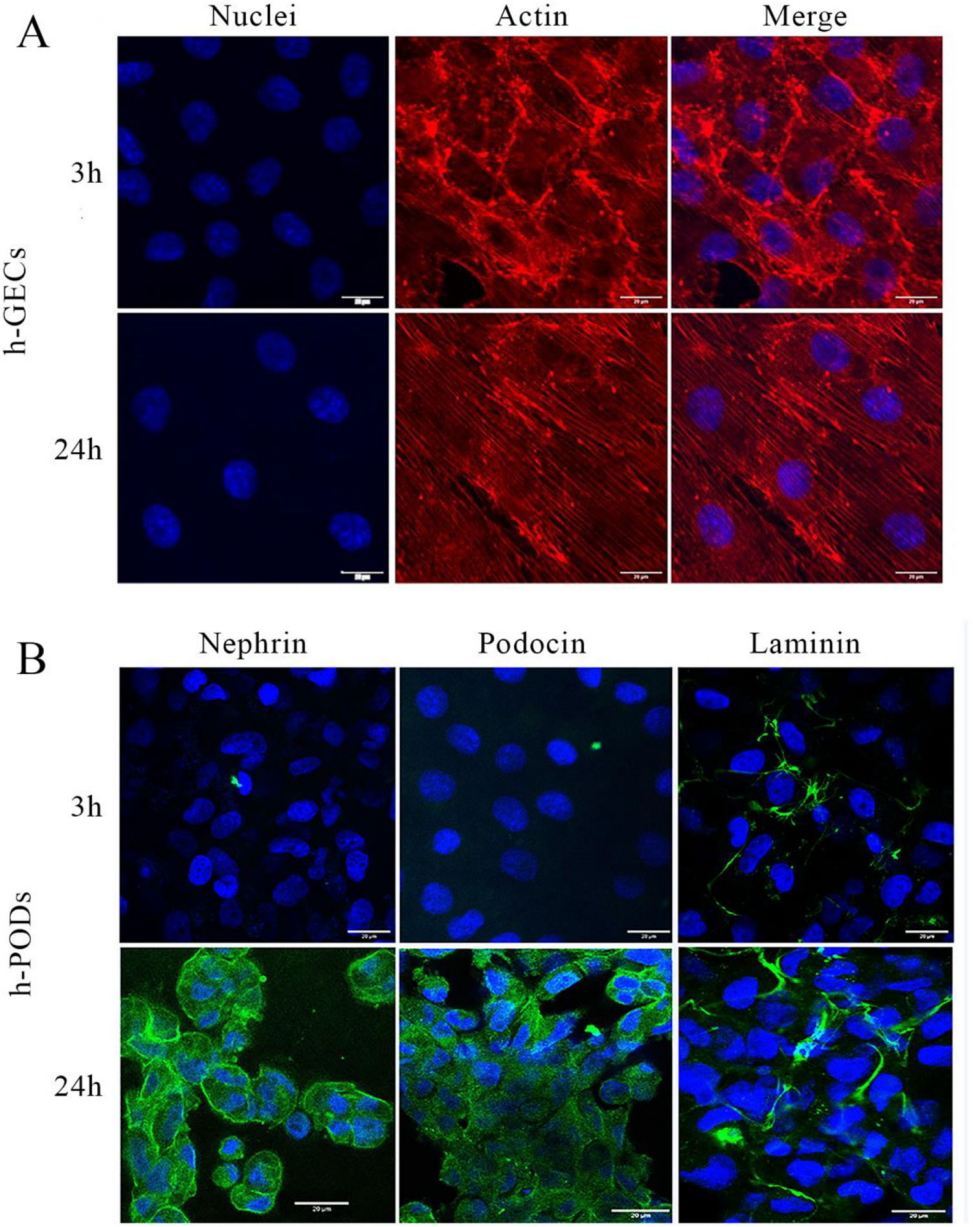
**A**, **B** Effect of flow on the 3D-glomerular dynamic model. Immunofluorescence of the co-culture of h-GEC (**A**) and h-PODs (**B**) submitted to perfusion for 3 h and 24 h. Membrane were then fixed and h-GECs (**A**) were stained for actin (in red) and nuclei (in blue). Podocytes were stained for Nephrin and Podocin (in green), laminin (in red) and nuclei (in blue). The images were acquired with a Leica SP5 confocal microscope, 40× objective, scale bar: 20 µm

Endothelial cells in the dynamic system were able to modify their actin filaments in the direction of shear stress, in a physiologic fashion, after 3 and 24 h of flow (Fig. 4A). We also assessed the production of basal membrane components, such as laminin, required for the barrier's integrity and the podocyte phenotype. We found that in the 3D dynamic glomerulus during perfusion, h-PODs increased the expression of nephrin and podocin, proteins that compose the slit diaphragm, in a time-dependent manner (Fig. 4B). Moreover, the deposition of laminin produced by the glomerular cells was also increased during perfusion (Fig. 4B)*.*

### Tracking EV fate using the 3D-glomerular model

**Fig. 5:**
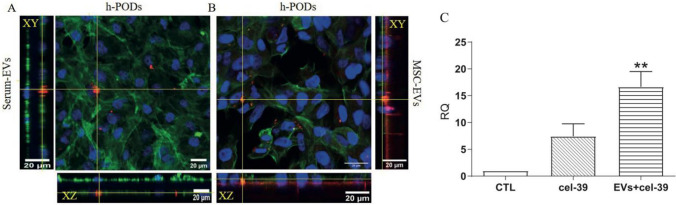
Internalization of labelled EVs carrying cel-miR-39 by h-PODs in the dynamic model. **A**, **B** Immunofluorescence of the h-PODs subjected to a dynamic perfusion for 24 h in the presence of DII labelled EVs from serum (Serum-EVs) and EVs from MSC (MSC-EVs). Incorporated EVs (in red) were detected in both conditions. Cells were co-stained for Actin (in green), nuclei (in blue). The images were made with Leica SP5 confocal, 40× objective (scale bar: 20 µm). Orthogonal sections were generated with ImageJ software. **C** Detection by qRT-PCR of the active transfer of cel-miR-39 by MSC-EVs (EVs+cel-39) in h-PODs after dynamic perfusion for 24 h. Cells were perfused for 24 h with cel-miR-39 (cel-39) or with culture medium (CTL) alone were used as control. In all the experiments, EVs+cel-39 and cel-39 alone were inserted into the endothelial compartment, and the presence of cel-39 internalized by h-PODs was then analyzed. RNU6B was used as normalizer. **p < 0.01 with respect to CTL

We speculated that the 3D model could be a helpful tool for tracking in a physiological context the dynamics of EV passage from the endothelial to the podocyte compartment. For this purpose, we tested the capability of co-cultured podocytes to internalize EVs of different origin under dynamic perfusion within the fluid circulating on the endothelial side. EVs from MSCs or from serum were labelled with the lipophilic DIl dye and electroporated with cel-miR-39, an exogenous miRNA from *C. Elegans*. The 3D-glomerular model was then perfused for 24 h with the cel-miR-39 enriched EVs (4 × 10^9^) or cel-miR-39 alone, and podocytes were analyzed for the uptake of EVs by immunofluorescence and for miR transfer by RT-PCR (Fig. 5). DII labelled EVs from both MSCs or serum could pass the glomerular barrier and target the co-cultured podocytes, and by confocal microscopy they were detectable within the cell body (Fig. 5A, B). Internalization of cel-miR-39 was also confirmed by qRT-PCR (Fig. 5C). Interestingly, EVs showed a strong delivery of cel-miR-39 to the co-cultured podocytes in respect to free cel-miR-39 (Fig. 5C).

### Development of glomerular damage using the 3D-glomerular model

**Fig. 6:**
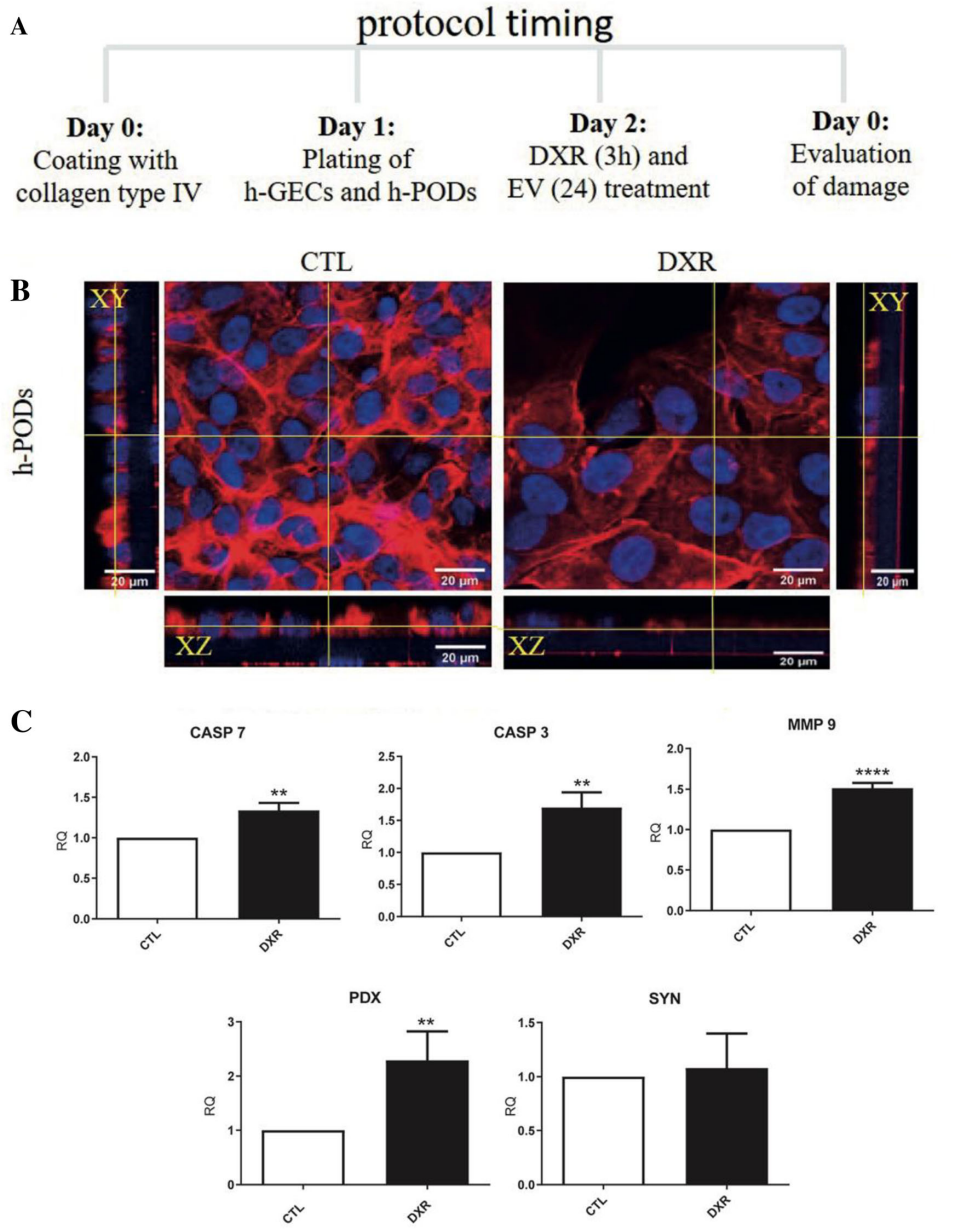
Effect of DXR treatment in the 3D-glomerular dynamic model. **A** Scheme of drug and regenerative EV administration in the 3D-glomerulus. **B** Immunofluorescence analysis of co-cultured h-PODs treated with DXR for 3 h followed by reperfusion for 24 h (DXR) or maintained in control medium (CTL). hPODs were stained for actin (in red) and nuclei (in blue). **C** qRT-PCR analysis of the cell damage markers Casp3, Casp7 and MMP9 and of the epithelial markers, PDX and SYN in control or DXR-treated hPODs. GAPDH was used as housekeeping gene. The results obtained were made comparable by setting the control (CTL) equal to 1. **p < 0.01, ***p < 0.001, ****p < 0.0001 with respect to CTL

To test the versatility of our system, we developed a model of glomerular damage induced by doxorubicin. A scheme of the protocol used is represented in Fig. 6A. Briefly, a doxorubicin enriched culture medium (0.5 µg/mL) was added within the endothelial compartment and maintained for 3 h. The stimulus was then removed, and basal culture medium was added in both endothelial and podocyte compartments for 24 h.

**Fig. 7:**
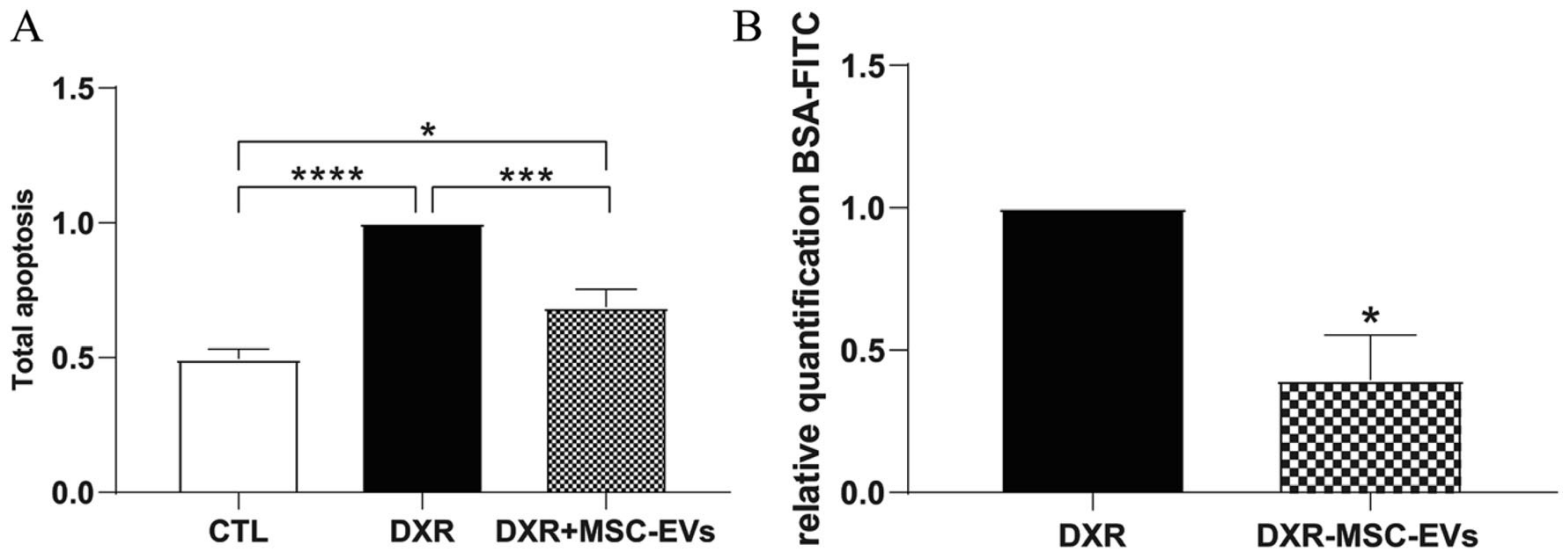
Protective effects of MSC-EVs on the 3D-glomerular dynamic model treated with DXR. **A** Evaluation of the apoptosis rate in co-cultured h-PODs treated with DXR for 3 h and then stimulated with MSC-EVs (DXR+MSC-EVs) or medium alone (DXR) for 24 h. Untreated co-cultured h-PODs (CTL) were used as negative control. The results obtained were made comparable by setting the control (CTL) equal to 1. *p < 0.05, ***p < 0.001, ****p < 0.0001 respectively. **B** Permeability test in DXR treated co-cultures in the absence (DXR) or presence of MSC-EVs (DXR+MSC-EVs). Perfusion was calculated as a percentage of filtered FITC-BSA (FITC-BSA solution = 1 mg/mL as 100% fluorescence); then normalized to DXR = 1. *p < 0.05 with respect to DXR

Doxorubicin treatment drastically affected the podocyte cytoskeleton as detected by actin staining. The treatment was able to create discontinuities in the epithelial layer, narrowing the podocyte body (Fig. 6B). Moreover, the expression of markers of apoptosis such as caspase 3, 7 (CASP3 and CASP7) and metalloproteinase 9 (MMP9) was significantly increased in doxorubicin treated h-PODs in comparison with co-cultured h-PODs only subjected to flow for 24 h (Fig. 6C). The expression of podocalyxin (PDX), a protein highly involved in filtration integrity maintenance [27], was also induced during the drug treatment, suggesting the capability of podocytes to activate a damage response in the 3D glomerular model. Finally, doxorubicin treatment for 24 h induced relevant apoptosis in podocytes co-cultured in the 3D-glomerular model in respect to the control untreated cells (Fig. 7A).

### Regenerative properties of MSC-EVs in the 3D-glomerular damage model

We finally tested the potential of MSC- EVs to protect podocytes after the doxorubicin treatment, EVs (4 × 10^9^) or medium alone were added to the endothelial circuit for 24 h following doxorubicin damage, in a condition of fluid recirculation both in the upper and in the lower part of the chamber. MSC- EV treatment significantly reduced the number of apoptotic cells as compared to doxorubicin treatment alone (Fig. 7A). Furthermore, the permeability test showed an improvement in the GFB integrity by MSC-EVs, with a consequent decrease in the passage of albumin through the barrier (Fig. 7B), indicating the ability of MSC-EVs to cross the glomerular filtration barrier and protect podocytes in a dynamic system.

## Discussion

Fluidic 3D models represent an innovative technology for preclinical* in vitro* research. This is especially true for organs in which hemodynamics plays a relevant role in organ homeostasis such as liver, heart and kidney. We here propose a simplified model mimicking a human glomerulus* in vitro*, composed of the three functional layers, the endothelial cells, the glomerular basal membrane and the podocytes, cultured in the presence of continuous perfusion. Taking advantage of these models, we showed EV passage from the endothelial to the podocyte compartment under flux and their therapeutic effect in a model of drug injury.

Glomerular hemodynamic depends upon integrated mechanical forces (including filtration pressure, flow, shear force, and traction force), which are fundamental for signalling and cell communication pathways [28]. Changes in ultrafiltrate flow underlie the pathogenesis of many kidney diseases [29]. In this study, the use of a constant flow of 80 µl/min allowed to mimic the physiological conditions of the glomerulus. This may be relevant not only for the endothelium but also for the podocyte layer, as shown by the increased expression of several podocyte proteins involved in the control of filtration after flow. It was demonstrated that during the ultrafiltrate passage within the Bowman's space and directly through the slit diaphragm, the flow of fluid applies tangential forces and increases shear stress on the podocyte body and foot processes [30]. Moreover, our 3D glomerular model could functionally block the passage of fluorescence-labelled serum albumin from the endothelial to the podocyte compartment, allowing us to assess albumin passage, universally recognized as an index of glomerular damage [31].

The passage of EVs present in the circulation toward the podocyte compartment and eventually to the ultrafiltrate is debated. Considering the small size (6 nm in the healthy state) of membrane‐pores of the glomerular filtration barrier and basement membrane of the kidney, a direct filtration of serum EVs appears unlikely and possible only in disease conditions [32–34]. However, the fenestrations of the endothelial layer appear of up to 100 nm in size, allowing EV penetration into the basal membrane [35]. In addition, mechanisms of transcytosis can be prospected. From a therapeutic point of view, biodistribution experiments of labelled i.v. injected EVs clearly showed renal localization of EVs in kidney injury models [22]. However, few studies detected podocyte localization, mainly in cell cultures [36]. We found that EVs added to the endothelial compartment of the 3D glomerulus could target podocytes and transfer an exogenous miRNA. However, we cannot exclude that in our system, increased permeability of the layers could occur.

Several groups have investigated the therapeutic potential of different EV sources in kidney diseases [37]. Interestingly, we also found that EVs administered to the endothelial compartment effectively restored podocyte viability [ref]. EVs from bone marrow-derived MSCs (MSC-EVs) have been proposed as a promising source of pro-regenerative molecules in a different model of AKI in mice [18, 38]. In this context, MSC-EV administration in our model significantly reduced doxorubicin-dependent apoptosis in co-cultured podocytes and subsequently the passage of albumin throughout the system by preserving the glomerular filtration barrier. The same effect of administered MSC-EVs on podocyte apoptosis was previously demonstrated using adipose-derived stem cell EVs in a model of type-2 diabetes in mice [39].

In conclusion, using an innovative millifluidic device that can easily mimic the human glomerular filtration barrier, we could trace EVs across the barrier and confirm their therapeutic effect on the podocyte compartment. This study opens the possibility to use this device in the future in a multi-organ circuit for EV metabolism studies.

## References

[1] Traub O, Berk BC (1998). Laminar shear stress: mechanisms by which endothelial cells transduce an atheroprotective force. Arterioscler Thromb Vasc Biol.

[2] Kim S, Takayama S (2015). Organ-on-a-chip and the kidney. Kidney Res Clin Pract.

[3] Van Norman GA (2019). Limitations of animal studies for predicting toxicity in clinical trials: is it time to rethink our current approach?. JACC Basic Transl Sci.

[4] Reiser J, Altintas MM (2016). Podocytes. F1000Research.

[5] Leung N, Drosou ME, Nasr SH (2018). Dysproteinemias and glomerular disease. Clin J Am Soc Nephrol.

[6] Richard Kitching A, Hutton HL (2016). The players: cells involved in glomerular disease. Clin J Am Soc Nephrol.

[7] Ashammakhi N, Wesseling-Perry K, Hasan A, Elkhammas E, Zhang YS (2018). Kidney-on-a-chip: untapped opportunities. Kidney Int.

[8] Zhou M, Zhang X, Wen X, Wu T, Wang W, Yang M, Wang J, Fang M, Lin B, Lin H (2016). Development of a functional glomerulus at the organ level on a chip to mimic hypertensive nephropathy. Sci Rep.

[9] Wang L, Tao T, Su W, Yu H, Yu Y, Qin J (2017). A disease model of diabetic nephropathy in a glomerulus-on-a-chip microdevice. Lab Chip.

[10] Musah S, Mammoto A, Ferrante TC, Jeanty SSF, Hirano-Kobayashi M, Mammoto T, Roberts K, Chung S, Novak R, Ingram M, Fatanat-Didar T, Koshy S, Weaver JC, Church GM, Ingber DE (2017). Mature induced-pluripotent-stem-cell-derived human podocytes reconstitute kidney glomerular-capillary-wall function on a chip. Nat Biomed Eng.

[11] Grange S, Marabese B (2019). Stem cell-derived extracellular vesicles and kidney regeneration. Cells.

[12] Ranghino A, Bruno S, Bussolati B, Moggio A, Dimuccio V, Tapparo M, Biancone L, Gontero P, Frea B, Camussi G (2017). The effects of glomerular and tubular renal progenitors and derived extracellular vesicles on recovery from acute kidney injury. Stem Cell Res Ther.

[13] Choi M, Ban T, Rhim T (2014). Therapeutic use of stem cell transplantation for cell replacement or cytoprotective effect of microvesicle released from mesenchymal stem cell. Mol Cells.

[14] Jiang ZZ, Liu YM, Niu X, Yin JY, Hu B, Guo SC, Fan Y, Wang Y, Wang NS (2016). Exosomes secreted by human urine-derived stem cells could prevent kidney complications from type i diabetes in rats. Stem Cell Res Ther.

[15] Nagaishi K, Mizue Y, Chikenji T, Otani M, Nakano M, Konari N, Fujimiya M (2016). Mesenchymal stem cell therapy ameliorates diabetic nephropathy via the paracrine effect of renal trophic factors including exosomes. Sci Rep.

[16] Hassan MH, Ghobara M, Abd-Allah GM (2014). Modulator effects of meloxicam against doxorubicin-induced nephrotoxicity in mice. J Biochem Mol Toxicol.

[17] Conaldi PG, Bottelli A, Baj A, Serra C, Fiore L, Federico G, Bussolati B, Camussi G (2002). Human immunodeficiency virus-1 Tat induces hyperproliferation and dysregulation of renal glomerular epithelial cells. Am J Pathol.

[18] Bruno S, Grange C, Collino F, Deregibus MC, Cantaluppi V, Biancone L, Tetta C, Camussi G (2012). Microvesicles derived from mesenchymal stem cells enhance survival in a lethal model of acute kidney injury. PLoS ONE.

[19] Bruno S, Grange C, Deregibus MC, Calogero RA, Saviozzi S, Collino F, Morando L, Busca A, Falda M, Bussolati B, Tetta C, Camussi G (2009). Mesenchymal stem cell-derived microvesicles protect against acute tubular injury. J Am Soc Nephrol.

[20] Cavallari C, Ranghino A, Tapparo M, Cedrino M, Figliolini F, Grange C, Giannachi V, Garneri P, Deregibus MC, Collino F, Rispoli P, Camussi G, Brizzi MF (2017). Serum-derived extracellular vesicles (EVs) impact on vascular remodeling and prevent muscle damage in acute hind limb ischemia. Sci Rep.

[21] Pomatto MAC, Bussolati B, D’Antico S, Ghiotto S, Tetta C, Brizzi MF, Camussi G (2019). Improved loading of plasma-derived extracellular vesicles to encapsulate antitumor miRNAs. Mol Ther Methods Clin Dev.

[22] Grange C, Tapparo M, Bruno S, Chatterjee D, Quesenberry PJ, Tetta C, Camussi G (2014). Biodistribution of mesenchymal stem cell-derived extracellular vesicles in a model of acute kidney injury monitored by optical imaging. Int J Mol Med.

[23] Iampietro C, Bellucci L, Arcolino FO, Arigoni M, Alessandri L, Gomez Y, Papadimitriou E, Calogero RA, Cocchi E, Van Den Heuvel L, Levtchenko E, Bussolati B (2020). Molecular and functional characterization of urine-derived podocytes from patients with Alport syndrome. J Pathol.

[24] Zhou M, Zhang X, Wen X, Wu T, Wang W, Yang M, Wang J, Fang M, Lin B, Lin H (2016). Development of a functional glomerulus at the organ level on a chip to mimic hypertensive nephropathy. Sci Rep.

[25] Li M, Corbelli A, Watanabe S, Armelloni S, Ikehata M, Parazzi V, Pignatari C, Giardino L, Mattinzoli D, Lazzari L, Puliti A, Cellesi F, Zennaro C, Messa P, Rastaldi MP (2016). Three-dimensional podocyte–endothelial cell co-cultures: assembly, validation, and application to drug testing and intercellular signaling studies. Eur J Pharm Sci.

[26] Slater SC, Beachley V, Hayes T, Zhang D, Welsh GI, Saleem MA, Mathieson PW, Wen X, Su B, Satchell SC (2011). An in vitro model of the glomerular capillary wall using electrospun collagen nanofibres in a bioartificial composite basement membrane. PLoS ONE.

[27] Refaeli I, Hughes MR, Wong AKW, Bissonnette MLZ, Roskelley CD, Wayne Vogl A, Barbour SJ, Freedman BS, McNagny KM (2020). Distinct functional requirements for podocalyxin in immature and mature podocytes reveal mechanisms of human kidney disease. Sci Rep.

[28] Endlich N, Endlich K (2012). The challenge and response of podocytes to glomerular hypertension. Semin Nephrol.

[29] Chagnac A, Zingerman B, Rozen-Zvi B, Herman-Edelstein M (2019). Consequences of glomerular hyperfiltration: the role of physical forces in the pathogenesis of chronic kidney disease in diabetes and obesity. Nephron.

[30] Kriz W, Lemley KV (2015). A potential role for mechanical forces in the detachment of podocytes and the progression of CKD. J Am Soc Nephrol.

[31] Levey AS, Becker C, Inker LA (2015). Glomerular filtration rate and albuminuria for detection and staging of acute and chronic kidney disease in adults: a systematic review. JAMA - J Am Med Assoc.

[32] Longmire M, Choyke PL, Kobayashi H (2008). Clearance properties of nano-sized particles and molecules as imaging agents: considerations and caveats. Nanomedicine.

[33] Patrakka J, Lahdenkari AT, Koskimies O, Holmberg C, Wartiovaara J, Jalanko H (2002). The number of podocyte slit diaphragms is decreased in minimal change nephrotic syndrome. Pediatr Res.

[34] Erdbrügger U, Blijdorp CJ, Bijnsdorp IV, Borràs FE, Burger D, Bussolati B, Byrd JB, Clayton A, Dear JW, Falcón-Pérez JM, Grange C, Hill AF, Holthöfer H, Hoorn EJ, Jenster G, Jimenez CR, Junker K, Klein J, Knepper MA, Koritzinsky EH, Luther JM, Lenassi M, Leivo J, Mertens I, Musante L, Oeyen E, Puhka M, Royen ME, Sánchez C, Soekmadji C, Thongboonkerd V, Steijn V, Verhaegh G, Webber JP, Witwer K, Yuen PST, Zheng L, Llorente A, Martens-Uzunova ES (2021). Urinary extracellular vesicles: a position paper by the urine task force of the international society for extracellular vesicles. J Extracell Ves.

[35] Ndisang JF (2018). Glomerular endothelium and its impact on glomerular filtration barrier in diabetes: are the gaps still illusive?. Curr Med Chem.

[36] Hill N, Michell DL, Sheng Q, Pusey C, Vickers KC, Woollard KJ (2020). Glomerular endothelial derived vesicles mediate podocyte dysfunction: a potential role for miRNA. PLoS ONE.

[37] Corrêa RR, Juncosa EM, Masereeuw R, Lindoso RS (2021). Extracellular vesicles as a therapeutic tool for kidney disease: current advances and perspectives. Int J Mol Sci.

[38] Gatti S, Bruno S, Deregibus MC, Sordi A, Cantaluppi V, Tetta C, Camussi G (2011). Microvesicles derived from human adult mesenchymal stem cells protect against ischaemia-reperfusion-induced acute and chronic kidney injury. Nephrol Dial Transpl.

[39] Jin J, Shi Y, Gong J, Zhao L, Li Y, He Q (2019). Exosome secreted from adipose-derived stem cells attenuates diabetic nephropathy by promoting autophagy flux and inhibiting apoptosis in podocyte. Stem Cell Res Ther.

